# In Vivo Fluorescence Imaging Reveals the Promotion of Mammary Tumorigenesis by Mesenchymal Stromal Cells

**DOI:** 10.1371/journal.pone.0069658

**Published:** 2013-07-25

**Authors:** Chien-Chih Ke, Ren-Shyan Liu, Atsushi Suetsugu, Hiroaki Kimura, Jennifer H. Ho, Oscar K. Lee, Robert M. Hoffman

**Affiliations:** 1 AntiCancer, Inc., San Diego, California, United States of America; 2 Institute of Clinical Medicine, National Yang-Ming University, Taipei, Taiwan; 3 Department of Orthopaedics and Traumatology, Taipei Veterans General Hospital, Taipei, Taiwan; 4 Department of Surgery, University of California San Diego, San Diego, California, United States of America; 5 Department of Nuclear Medicine, National Yang-Ming University School of Medicine, Taipei, Taiwan; 6 National PET/Cyclotron Center, Department of Nuclear Medicine, Taipei Veterans General Hospital, Taipei, Taiwan; 7 Taiwan Mouse Clinic, National Comprehensive Mouse Phenotyping and Drug Testing Center, Taipei, Taiwan; 8 Graduate Institute of Clinical Medicine, Taipei Medical University, Taipei, Taiwan; 9 Center for Stem Cell Research, Taipei Medical University-Wan Fang Medical Center, Taipei, Taiwan; 10 Stem Cell Research Center, National Yang-Ming University, Taipei, Taiwan; Cincinnati Children's Hospital Medical Center, United States of America

## Abstract

Mesenchymal stromal cells (MSCs) are multipotent adult stem cells which are recruited to the tumor microenvironment (TME) and influence tumor progression through multiple mechanisms. In this study, we examined the effects of MSCs on the tunmorigenic capacity of 4T1 murine mammary cancer cells. It was found that MSC-conditioned medium increased the proliferation, migration, and efficiency of mammosphere formation of 4T1 cells *in vitro*. When co-injected with MSCs into the mouse mammary fat pad, 4T1 cells showed enhanced tumor growth and generated increased spontaneous lung metastasis. Using *in vivo* fluorescence color-coded imaging, the interaction between GFP-expressing MSCs and RFP-expressing 4T1 cells was monitored. As few as five 4T1 cells could give rise to tumor formation when co-injected with MSCs into the mouse mammary fat pad, but no tumor was formed when five or ten 4T1 cells were implanted alone. The elevation of tumorigenic potential was further supported by gene expression analysis, which showed that when 4T1 cells were in contact with MSCs, several oncogenes, cancer markers, and tumor promoters were upregulated. Moreover, *in vivo* longitudinal fluorescence imaging of tumorigenesis revealed that MSCs created a vascularized environment which enhances the ability of 4T1 cells to colonize and proliferate. In conclusion, this study demonstrates that the promotion of mammary cancer progression by MSCs was achieved through the generation of a cancer-enhancing microenvironment to increase tumorigenic potential. These findings also suggest the potential risk of enhancing tumor progression in clinical cell therapy using MSCs. Attention has to be paid to patients with high risk of breast cancer when considering cell therapy with MSCs.

## Introduction

Mesenchymal stromal cells (MSCs) are adult stem cells that possess multipotent differentiation potential. In addition to progenies of mesodermal lineages including osteoblasts, chondrocytes, adipose cells and muscle cells [1], MSCs are also able to trans-differentiate into endodermal lineages such as hepatocytes [2]. MSCs primarily reside within the bone marrow [3], but also can be isolated from umbilical cord blood, adipose tissue, adult muscle, and the dental pulp of deciduous baby teeth [4], [5]. Recently, it has been reported that MSCs have multiple effects on cancer progression. When MSCs are systemically injected into tumor-bearing animals, they specifically target tumors [6]-[8]. Factors such as stromal cell-derived factor 1 (SDF-1) and its receptor C-X-C chemokine receptor type 4 (CXCR-4), platelet-derived growth factor α (PDGF-α) and vascular endothelial growth factor (VEGF) may be involved in MSC targeting to tumors [9], [10]. The recruited MSCs within the tumor microenvironment (TME) may further differentiate into various types of cells, such as fibroblasts, pericytes and cancer-associated fibroblasts (CAFs) [11], [12] which influence cancer progression. MSCs also promote angiogenesis. Several growth factors and cytokines secreted by MSCs, such as VEGF, angiopoietin, Interleukin 6, Interleukin 8, transforming growth factor β (TGF-β), PDGF, bFGF, and FGF-7 may act on endothelial cells and directly contribute to tumor vessel formation [13]. Interaction of the chemokine CCL5 and its receptor CCR5 between MSCs and breast cancer cells, respectively, has been shown to enhance cancer cell motility, invasion and metastasis of breast cancer cells [14]. Moreover, MSCs enhanced *in vitro* mammosphere formation by breast cancer cells and reduced the latency time of *in vivo* tumor formation [15].

The use of fluorescent proteins for *in vivo* imaging enables cell behavior to be observed within a living subject. More importantly, the interaction between different types of cells can also be visualized by labeling each type of cell with a different colored fluorescent protein [16]. Using this approach, we previously generated a color-coded TME that allowed imaging of the interaction between cancer-associated fibroblasts (CAFs) and metastatic colon cancer in the liver [17]. In the present study, we used color-coded imaging to demonstrate how MSCs affect the gross tumor formation of breast cancer cells.

## Materials and Methods

### Cell Isolation and Culture

Isolation of mouse bone marrow-derived mesenchymal stromal cells was performed according to previously reported methods [18] with slight modifications. Briefly, hind tibiae and femurs of transgenic mice ubiquitously expressing GFP or RFP were removed after the animals were sacrificed. Both ends were cut and a marrow plug was flushed out with a 27-gauge needle connected to a syringe filled with complete medium. The marrow was washed with PBS twice and then cultured in DMEM (Thermo Fisher Scientific, Rockford, IL, USA) supplemented with 10% fetal bovine serum in a 37°C incubator. After 48 hours, unattached cells were removed and then the medium was changed regularly every 3 days. The mouse mammary cancer cell lines, 4T1 and JC, purchased from ATCC (Manassas, VA) and BCRC (Bioresource Collection and Research Centre, Hsinchu, Taiwan), respectively were cultured in RPMI-1640 medium (Thermal Fisher Scientific, Rockford, IL, USA) supplemented with 10% fetal bovine serum.

4T1 cells were retrovirally infected with a GFP- or RFP-expressing vector as described previously [19]–[22]. Briefly, a RetroXpress vector (CLONTECH Laboratories, Inc., Palo Alto. CA, USA), expressing either GFP or RFP, was used for virus production. Retroviruses were packaged using PT67, a NIH3T3-derived packaging cell line and then cultured in DMEM (Thermal Fisher Scientific, Rockford, IL, USA) supplemented with fetal bovine serum (Gemini Biological Products, Calabasas, CA, USA). At 20%-confluence, the 4T1 cells were cultured in a 1∶1 mixture of fresh medium and precipitated retroviral supernatant from PT67 cells for 72 h. Fluorescent protein-expressing cells were selected by culturing in medium containing G418, with stepwise concentration increases (from 400 to 1,000 µg/ml). In order to produce the MSC-conditioned medium (MSC-CM), medium used for culturing MSCs for 2 days was collected and kept at 4°C for one day if used immediately, or stored at −20°C for long-term use. The MSC-CM was used to culture 4T1 cells for subsequent experiments.

### *In vitro* Cell Proliferation and Colony Formation

To assess cell proliferation, 10^4^ 4T1 cells were seeded in 6-well plates and cultured in the presence of either regular medium or MSC-CM. At various time points after cell seeding, cells were released completely by trypsin and viable cells were identified by trypan blue exclusion and counted. To assess colony formation, each well of a 6-well plate was seeded with 500 4T1 cells and cultured in the presence of either regular medium or MSC-CM. After 6 days, the colonies were washed twice with ice-cold PBS and fixed with 3.7% paraformaldehyde for 5 min. Crystal violet dissolved in distilled water [0.05% (w/v)] was used to stain the pre-fixed colonies for 20 min. After rinsing with tap water and air-drying, the number of colonies was counted. Only colonies consisting of more than 50 cells were counted.

### *In vitro* Wound Healing Assay

4T1 cells were seeded in 6-well plates and cultured with RPMI-1640 medium. When the cells reached confluence, gaps were introduced by scratching, using a micro-pipette tip. After two washings with PBS to remove detached cells and debris, the culture medium was replaced by either MSC-CM, regular medium, or a 1∶1 mixture of MSC-CM and regular medium. Gap closure was monitored and photographed at different time points after scratches were made using an Olympus IX-71 microscope equipped with a Hamamatsu color CCD camera (Hamamatsu Photonics, Hamamatsu, Japan). The size of the gaps was measured using Image Pro Plus software.

### *In vitro* Mammosphere Formation

4T1 cells were cultured in regular medium or MSC-CM for 1 week with a change of medium every 2 days. After trypsinization, the presence of a single-cell suspension was confirmed by microscopy. Cells were counted and seeded at 2000 cells/100 µl/well in a 96 well ultra-low-attachment plate (Corning Incorporated, Corning, NY, USA) and cultured with DMEM-F12 medium supplemented with 2% B27 (Gibco-Invitrogen, Carlsbad, CA, USA), 20 ng/ml EGF, 20 ng/ml bFGF, 0.5 mg/ml, hydrocortisone and 5 mg/ml insulin. The number of mammospheres present in each well was counted after culture for 1 week.

### Messenger RNA Expression Profiling

GeneChip Mouse genome 430 2.0 arrays (Affymetrix, Santa Clara, CA, USA), containing probe sets for >45,000 characterized genes and expressed sequence tags, were used. Sample labeling and processing, GeneChip hybridization, and scanning were performed according to Affymetrix protocols. Briefly, total RNA was extracted using Trizol reagent (Invitrogen, Carlsbad, CA, USA). Next, cDNA synthesis, fragmentation, hybridization, washing, staining and scanning were performed at the National Research Progress for Genomic Medicine Microarray and Gene Expression Analysis Core Facility, National Yang-Ming University VYM Genome Research Center.

### Ethics Statement

All animal studies were approved by the AntiCancer Inc. Institutional Animal Care and Use Committee (IACUC) and conducted in accordance with the principles and procedures outlined in the National Institutes of Health Guide for the Care and Use of Animals under Assurance No. A3873-01. Animals were kept within a barrier under HEPA filtration. Mice were fed autoclaved laboratory rodent diet (Tecklad LM-485, Western Research Products, Orange, CA, USA). All surgical procedures were performed under anesthesia with an intramuscular injection of 100 µl of a ketamine mixture (10 µl ketamine HCL, 7.6 µl xylazine, 2.4 µl acepromazine maleate, and 10 µl PBS). Euthanasia was achieved either by intraperitoneal injection of an overdose of ketamine mixture or by 100% carbon dioxide inhalation, followed by cervical dislocation.

### Tumor Growth

Six-week-old female nude mice were used for the tumor growth study. 5×10^5^ 4T1 cells alone or premixed with an equal amount of MSCs were injected into the mammary fat pad of nude mice. Tumor volume (L×W×H×0.52 mm^3^) was measured twice a week using calipers for 3 weeks. The mice were then euthanized by injection of an overdose of the ketamine mixture when the tumor burden exceeded 10 mm in diameter or when they exhibited significant morbidity.

### Spontaneous Lung Metastasis

In order to image spontaneous lung metastasis, 5×10^5^ GFP-expressing 4T1 cells alone or premixed with an equal amount of MSCs were injected into the mammary fat pad of mice. After 3 weeks tumor growth, the mice were sacrificed with an overdose of ketamine mixture. After confirming the death of each mouse, the entire lungs of each mouse were excised, washed with PBS and separated into the various lobes. GFP-fluorescent colonies within each lobe were examined and imaged using an Olympus OV100 Imaging System (Olympus Corp., Tokyo, Japan) with a constant exposure time and offset.

### Tumorigenesis Assay

Either five, ten, or one hundred RFP-4T1 cells were injected subcutaneously into the mammary fat pad, either alone or with 2×10^5^ GFP-expressing MSCs. Each group consisted of at least five mice. Tumor incidence was recorded for up to 2 months and imaged using the OV-100 imaging system (Olympus). Mice were euthanized when the tumor burden exceeded 10 mm in diameter. To image the process of orthotopic mammary tumor formation, a reversible skin flap was raised according to the method described in [23]. Briefly, mice were anesthetized with a ketamine mixture via subcutaneous injection. An arc-shaped incision was made in the thoracic and abdominal skin. The subcutaneous connective tissue was separated to free a skin flap without injuring the vessels. The skin flap was spread and fixed on a flat stand. The mammary fat pad was imaged where the cells were injected with the OV-100 at various magnifications over time.

### Immunostaining and TUNEL Assay

Tumors were established by orthotopic implantation of 5×10^5^ 4T1 cells alone or with equal amount of MSCs as described above. Tumors were excised and immediately soaked in 30% sucrose (in PBS) at 4°C overnight. For cryosectioning, tissues were embedded in OCT compound and stored at −80°C. The sectioned tissue slices were blocked and permeabilized by blocking buffer (5% FBS and 0.03% Triton X 100 in PBS) for 1 hour. Immunofluorescence staining was performed by incubating tissue samples with primary antibodies (diluted in PBS containing 5% PBS) at 4°C overnight. After rinsing with PBS, tissue samples were incubated with corresponding secondary antibodies at room temperature for 1 hr followed by DAPI counterstaining. Samples were viewed and imaged by fluorescence microscopy. Antibodies used in this study included rat anti-CD31 monoclonal Ab (1∶100, ab7388, Abcam); rabbit anti-Ki67 polyclonal Ab (1∶100, ab15580, Abcam); DyLight^TM^488-conjugated goat anti-rabbit Ab (1∶500, 112-175-062, Jackson ImmunoResearch); and Cy^TM^5-conjugated goat anti-rat Ab (1∶500, 111-485-003, Jackson ImmunoResearch).

## Results

### MSCs Enhance 4T1 Cancer Cell Proliferation and Tumor Growth

**Figure 1:**
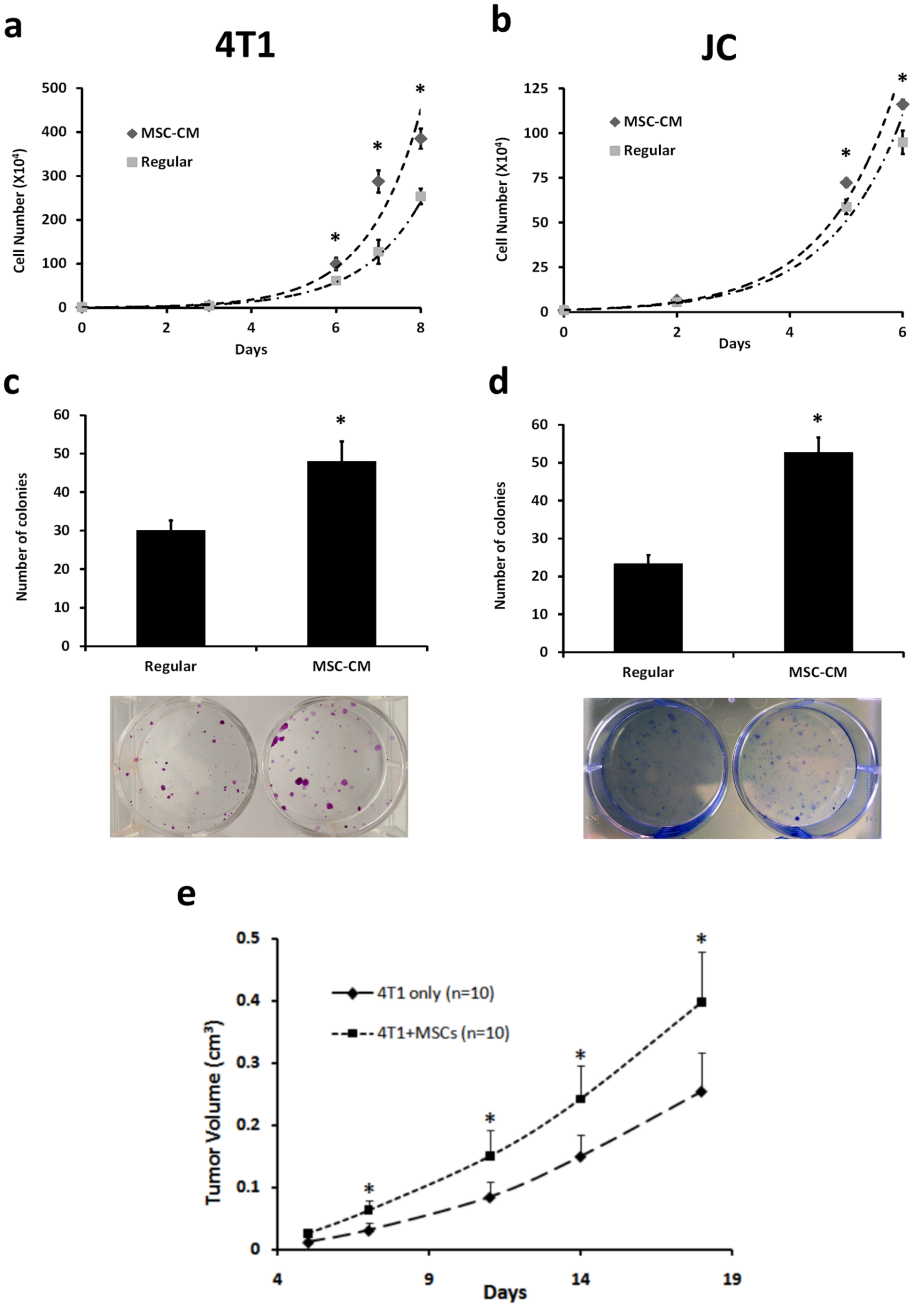
MSC enhances 4T1 cell proliferation and tumor growth. (a,b) To assess cell proliferation, 4T1 or JC cells were cultured in RPMI-1640 medium or MSC-CM with the medium changed every 2 days. Cells were counted from 6 wells at each time point after culture at the indicated time points. Cells cultured in CM proliferated more than those cultured in regular medium. (c, d) To carry out the colony forming assay, 500 4T1 or JC cells were seeded and cultured using RPMI-1640 medium or MSC-CM. The colonies were stained with crystal violet after being cultured for 6 days. The cells grown using MSC-CM showed greater potential to form colonies. (c) 5×10^5^ GFP-expressing 4T1 cells alone or premixed with an equal amount of RFP-expressing MSCs were injected subcutaneously into the mammary fat pad of nude mice. Each group consisted of ten mice. Tumor growth was measured twice a week and the data are shown as the mean ± SD. Tumors derived from a mixture of 4T1 cells and MSCs had significantly enhanced growth compared to tumors derived from 4T1 cells alone. All data are shown as the mean ± SD. “*” represents a significant difference of *p*<0.05.

To determine whether MSCs influence cancer cell proliferation, cells were counted at various time points after 4T1 or JC cells were cultured in regular medium or MSC-CM. Cells cultured in MSC-CM proliferated more than cells cultured in regular medium (Fig. 1a and 1b, p<0.05). In addition, using the colony-forming assay, cells cultured in MSC-CM formed more colonies than those in regular medium (Fig. 1c and 1d, p<0.05).

**Figure 2:**
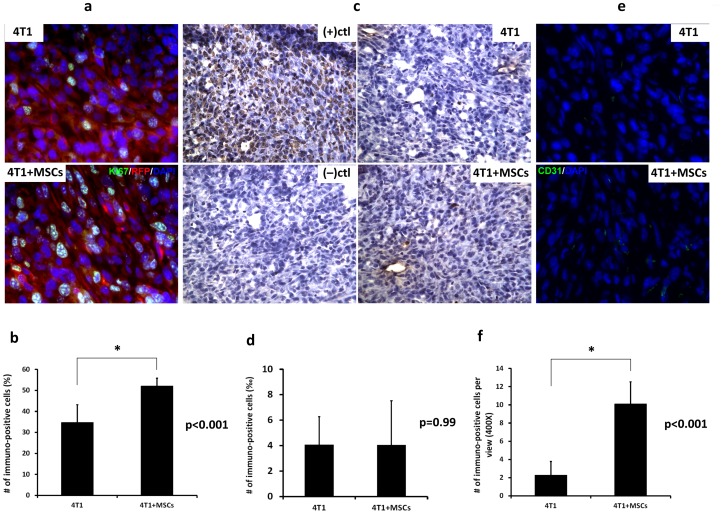
Tumor immunostaining for proliferation, apoptosis and angiogenesis. 5×10^5^ GFP-expressing 4T1 cells alone or premixed with an equal amount of RFP-expressing MSCs were injected subcutaneously into the mammary fat pad of nude mice. Tumors were excised for subsequent sectioning and immunostaining. Tumor sections from a 4T1 tumor or a 4T1+MSCs tumor were stained with antibody raised against Ki67 (a), CD31 (e) or subjected to TUNEL assay (c). (b, d and f) Quantification of Ki67, CD31 and TUNEL staining of tumor sections from a 4T1 tumor or a 4T1+MSCs tumor. Data represent mean values ±SD (n = 3). “*” represents a significant difference of *p*<0.001.

In order to investigate the effect of MSCs on tumor growth, 4T1 cells alone or pre-mixed with MSCs were injected into the mammary fat pad of mice and tumor volume was measured. On day 18, tumors formed from 4T1+MSC cell co-transplantation were 1.5-fold larger than the tumors derived from 4T1 cells alone. There was a significant difference in tumor volume between these two groups from day 7 after cell implantation (Fig. 1e, *p<0.02). Immunostaining of tumor sections also showed that the frequency of Ki67 expression was significantly higher in 4T1 cancer cells derived from 4T1+MSC tumors than those from 4T1 cells (Fig. 2a and 2b). These results indicated that co-implantation with MSCs resulted in the increase of proliferation in 4T1 cells and tumor-growth enhancement.

### MSCs Promote Cancer Cell Migration *in vitro* and Lung Metastasis *in vivo*

**Figure 3:**
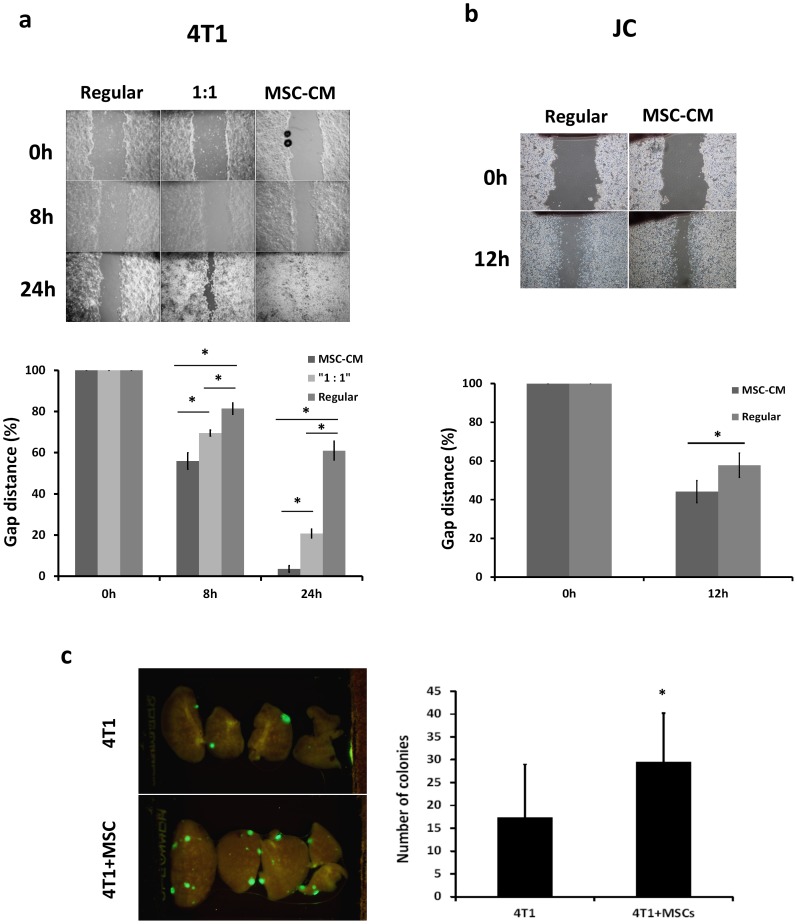
MSC-CM enhances 4T1 cell migration and lung metastasis. (a,b) Representative photomicrographs of the gaps of 4T1 and JC cells for each culture condition at different time points after scratching in a wound healing assay (upper). Quantification of wound-healing was assessed by measuring gap distance (lower). The cells showed a higher migratory ability when cultured in MSC-CM compared to cells cultured in RPMI-1640 medium. Data are presented as the percentage change in gap distance relative to 0 hr. Each bar shows mean ± SD of six measurements of each gap. Asterisk indicates a significant difference using the Student’s *t* test. (*p<0.05) (c) For the in vivo lung metastasis assay, 5×10^5^ GFP-expressing 4T1 cells alone, or premixed with an equal number of MSCs, were injected into the mammary fat pad. Each group contained at least nine mice. Three weeks later, the mice were sacrificed and their lungs excised and then imaged using the Olympus OV100 Imaging System with a constant exposure time and offset. Representative fluorescence images of the various lobes derived from the same lung. Lung tissue was excised from mice bearing 4T1 (right panel, n = 10) or 4T1+MSCs (left panel, n = 9) tumors. (d) Calculated number of GFP-fluorescent lung colonies. The number of colonies present in five lobes per lung, from both lungs was counted. MSCs promote spontaneous lung metastasis of orthotopic 4T1 tumors. Data are presented as mean ± SD with a significant difference (*p* = 0.03).

To assess the influence of MSCs on cancer cell migration, 4T1 or JC cells cultured in MSC-CM or regular medium were subjected to a wound-healing assay. Higher migration ability was observed when cancer cells were cultured in MSC-CM compared to regular medium (Fig. 3a and 3b) (*p<0.001). For 4T1 cells, the average gap distance in regular medium and MSC-CM narrowed to 81% and 56%, respectively, at 8 hr; and to 61% and 4%, respectively, at 24 hr (Fig. 3a). A similar effect was shown in JC cells, with the gap narrowed to 57% and 44%, respectively, of the original size at 12 hr when cells were in regular medium and MSC-CM, respectively (Fig. 3b).

MSCs also enhanced spontaneous metastasis of 4T1 tumors. We injected 5×10^5^ GFP-expressing 4T1 cells alone or pre-mixed with 5×10^5^ MSCs into the mammary fat pad of nude mice. GFP-expressing colonies in lung tissue were counted 3 weeks later. The tumors formed by co-injection gave rise to 29.6±11.5 spontaneous lung metastatic colonies, compared to 17.4±10.7 lung colonies produced by 4T1 cells alone (p = 0.03) (Fig. 3c).

### MSCs Promote Mammosphere Formation and Tumorigenesis

**Figure 4:**
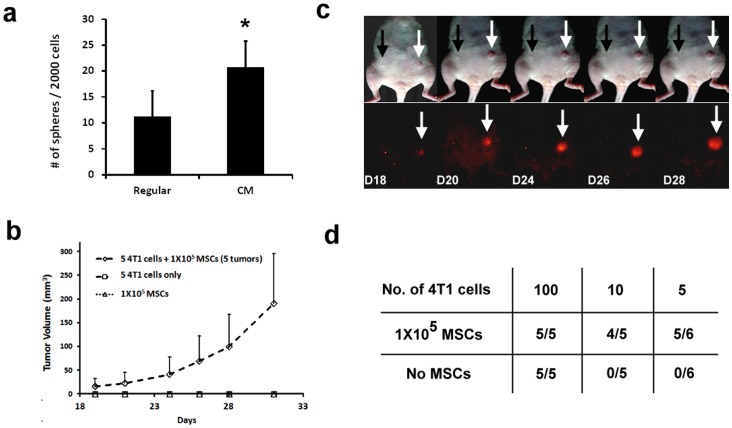
MSCs enhance 4T1 mammosphere formation and tumorigenicity. For the mammosphere formation assay, 4T1 cells were cultured in regular medium or MSC-CM for 1 week. After typsinization, a single-cell suspension was confirmed by microscopic observation. The cells were then seeded at 2000 cells/100 µl/well into 96 well ultra-low-attachment plates and cultured in mammosphere-forming medium. The number of spheres formed was counted after 1 week incubation. (a) Number of spheres formed from 2000 cells pre-cultured with RPMI-1640 or MSC-CM and counted from five wells. Cells pre-cultured in MSC-CM formed mammospheres more efficiently than those cultured in RPMI-1640 medium (*p* = 0.03). Data are presented as the mean ± SD. To evaluate the *in vivo* tumorigenic potential of 4T1 cell, a total of either 5, 10 or 100 RFP-expressing 4T1 cells alone or premixed with 1×10^5^ GFP-expressing MSCs, were injected into the mammary fat pad. Tumor incidence was observed and imaged. (b) Representative image of tumorigenesis. The white and black arrows indicate the sites where 4T1 cells mixed with MSCs or 4T1 cells alone were injected, respectively. Tumor growth was recorded and is shown in (b). (c) Summary of tumor formation when analyzed using limiting dilution.

Tumor sphere formation may depend on self-renewal of cancer stem cells and correlates with in vivo tumorigenic capacity. To determine whether MSCs influence sphere-forming ability, 4T1 cells were pre-cultured in either regular medium or MSC-CM for 1 week and then subjected to a mammosphere forming assay. 4T1 cells pre-cultured with MSC-CM formed mammospheres more efficiently than cells pre-cultured in regular medium (Fig. 4a).

We further investigated the effect of MSCs on 4T1 tumorigenesis *in vivo.* Either 100, 10 or 5 RFP-expressing 4T1 cells alone or pre-mixed with 1×10^5^ GFP-expressing MSC cells were injected subcutaneously into the mammary fat pad. One hundred 4T1 cells gave rise to tumors whether or not co-injected with MSCs. However, when either five or ten cells were injected, 4T1 cells formed tumors only when MSCs were present (Fig. 4). MSCs implanted alone did not result in tumor formation (Fig. 4b). Longitudinal *in vivo* fluorescence imaging demonstrated that during tumor formation (D18 to D28), only the RFP signal from the 4T1 cells was observed and not the GFP signal from MSCs, which supports the hypothesis that MSCs undergo no or negligible proliferation during the process of 4T1 tumorigenesis (Fig. 4c). These results clearly showed that 4T1 cells increased their ability of self-renewal and tumorigenic potential after modulation by MSCs.

### *In vivo* Imaging of MSC-4T1 Cell-cell Interaction

**Figure 5:**
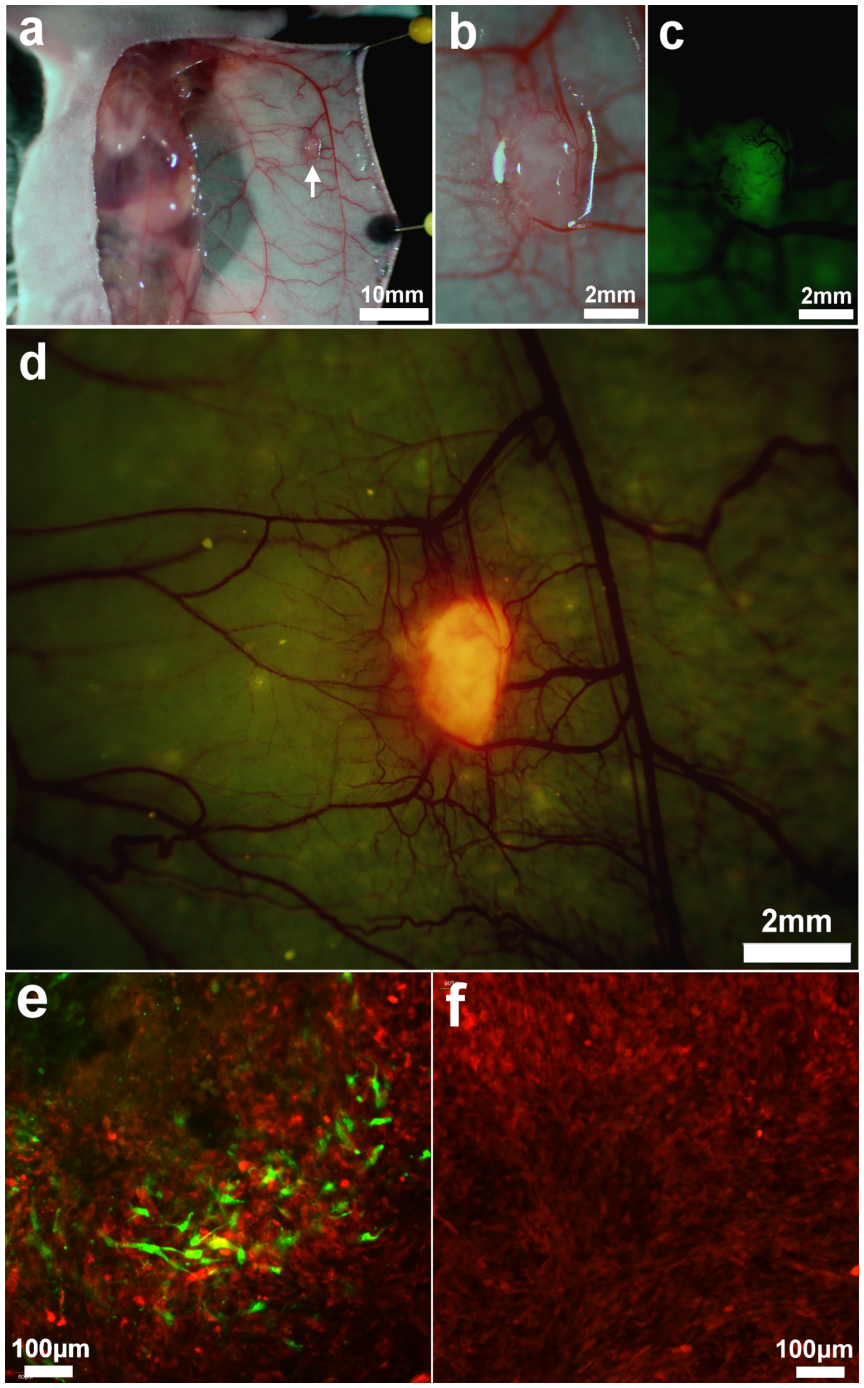
*In vivo* imaging of MSC-4T1 cell-cell interaction. GFP-expressing MSCs and RFP-expressing 4T1 cells (5×10^5^ each) were co-implanted into the mouse thoracic mammary fat pad. (a) 5 days after implantation, an arc shape incision was made in the abdominal skin. A skin flap was lifted above the mammary fat pad and imaged with the OV-100. White arrow indicates the location of tumor. (b–d). GFP and RFP signals were detected from the tumor which was surrounded by a dense vascular network. (e) The excised tumor was cross-sectioned and imaged using the Olympus IV100. GFP and RFP-expressing cells were observed within the tumor. (f) On day 11, GFP-MSCs were rarely observed within the tumor, suggesting MSCs were not proliferating during the growing of tumor.

To investigate the cellular interaction between 4T1 cell and MSCs *in vivo*, RFP-expressing 4T1 cells and GFP-expressing MSCs were co-implanted into the mouse mammary fat pad using 1×10^5^ of each cell type. One week after implantation, an arc-shape incision was made into the abdominal skin. The skin flap was raised above the mammary fat pad and imaged with the OV-100. At 1∼2 mm diameter, the tumor was visualized close to the epigastric cranialis vein, which had a thickened diameter. A dense vessel complex formed at the site of the tumor. Under fluorescence-light excitation, the tumor emitted both GFP and RFP signals from MSCs and 4T1 cells, respectively (Fig. 5a∼c). A cross-section of an excised tumor was immediately imaged using the Olympus IV100 laser-scanning microscope. GFP-expressing MSCs appeared fibroblastic with multiple protrusions which were dispersed among the RFP-expressing 4T1 cells within the tumor. The number of GFP-expressing MSCs was much lower than the RFP-expressing 4T1 cells, even though both types of cells were co-implanted with equal numbers (Fig. 5e). On day 11, when tumor volume was approximately 100 mm^3^, GFP-MSCs could rarely be seen (Fig. 5f) within the tumor, suggesting that the MSCs were not proliferating.

**Figure 6:**
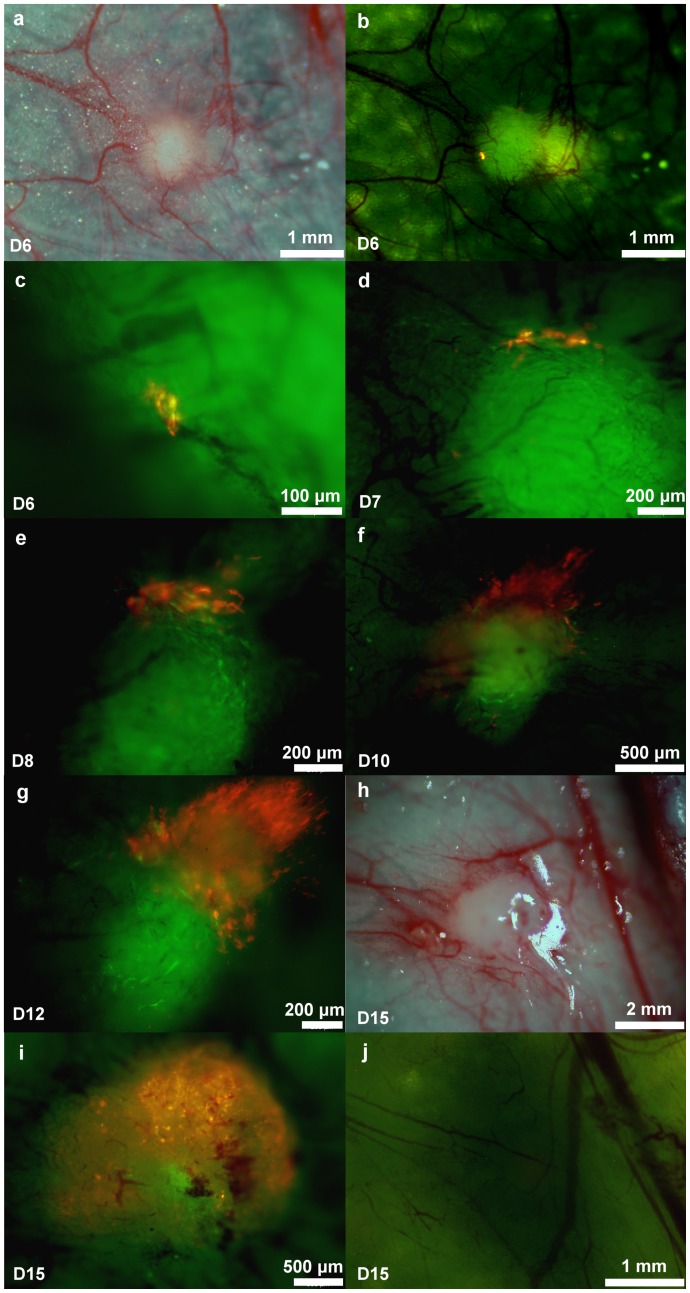
*In vivo* imaging of 4T1 tumorigenesis. Ten RFP-expressing 4T1 cells were injected alone or co-injected with 1×10^5^ GFP-expressing MSCs. An arc-shaped incision was made in the thoracic and abdominal skin and imaged with the OV-100 over time. (a∼k) Tumor initiation by ten 4T1 cells in the presence of MSCs was monitored. (l) No tumor incidence was found when 10 4T1 cells without MSCs were injected.

The process of 4T1 tumor initiation in the presence of MSCs was also imaged. Ten RFP-expressing 4T1 cells and 1×10^5^ GFP-expressing MSCs were co-implanted into the mouse mammary fat pad. On day 6 after injection, a GFP fluorescence-emitting MSC spherical mass was observed. Numerous tiny blood vessels stretched from outside the vascular trunk into the mass and formed an initial vessel network. Only a low RFP signal was detected within the mass. At higher magnification, approximately 6 RFP-expressing 4T1 cells were imaged (Fig. 6a∼c). On day 7, more RFP-expressing cells were observed within the sphere (Fig. 6d). After day 8, the number of 4T1 cells increased, and there was significant proliferation of these cells by day 15 (Fig. 6e∼k). When 10 RFP-expressing 4T1 cells were implanted alone, no cells were found later at or near the area of injection (Fig. 6l).

### Gene Expression Array Analysis and Immunochemistry

Comparing the relative gene expression levels of 4T1 cells which have been co-cultured with MSCs to 4T1 cells alone, 1270 genes that showed a change greater than two-fold were identified. A total of 684 upregulated genes were analyzed using gene ontology clustering based on DAVID Bioinformatics Resources (http://david.abcc.ncifcrf.gov). The clustered upregulated functional groups included genes encoding proteins related to the positive regulation of apoptosis (n = 22), the negative regulation of apoptosis (n = 14) (Table 1), the positive regulation of proliferation (n = 16), and the negative regulation of proliferation (n = 14) (Table 2). These data suggest that co-culture with MSCs induces opposing effects on both 4T1 cell proliferation and apoptosis at the gene expression level. Despite the complex gene expression pattern induced by MSCs, 4T1 cells or tumors showed enhanced cell proliferation and tumor growth *in vitro* and *in vivo* (Fig. 1 and Fig. 2a). Apoptosis was also assessed in tumors derived from implantation of 4T1 with or without MSCs. The result showed no difference between the two groups (Fig. 2c and 2d), suggesting that MSC-induced upregulation of pro-apoptotic genes did not trigger cell apoptosis. Among the upregulated genes, we identified several oncogenes (Ets2, Fyn, Fos, Rab30 and Src), various tumor markers (Cyp1b1, Gpa33, Cd47, Fam129a, st7l, chka, Lgalsbp, Antxr1 and Ly6a) and other genes related to tumor promotion (Cxcl10, Trim25, Grn, Foxc2, Bmp7 and Irs1) (Table 3). These upregulated genes help to partly explain enhanced tumor growth, despite the fact that the 4T1 cells also express higher level of genes related to apoptosis and negative regulation of proliferation. In summary, the gene expression profile of the 4T1 cells suggests that MSCs are able to induce multiple effects on 4T1 cells, including some that oppose each other.

**Table 1:** Cluster of genes involved in the positive and negative regulation of apoptosis.

ID	Gene Title	Gene Symbol	Fold Change
**Positive Regulation of Apoptosis**			
1423315_at	BCL2 binding component 3	Bbc3	2.639015822
1418901_at	CCAAT/enhancer binding protein (C/EBP), beta	Cebpb	2.639015822
1417516_at	DNA-damage inducible transcript 3	Ddit3	7.464263932
1460251_at	Fas (TNF receptor superfamily member 6)	Fas	5.278031643
1418242_at	Fas-associated factor 1	Faf1	2
1416029_at	Kruppel-like factor 10	Klf10	3.482202253
1450918_s_at	Rous sarcoma oncogene	Src	2
1435479_at	bone morphogenetic protein 7	Bmp7	3.031433133
1449591_at	caspase 4, apoptosis-related cysteine peptidase	Casp4	9.18958684
1426955_at	collagen, type XVIII, alpha 1	Col18a1	2.143546925
1437119_at	endoplasmic reticulum (ER) to nucleus signalling 1	Ern1	3.482202253
1434831_a_at	forkhead box O3	Foxo3	2.143546925
1419665_a_at	nuclear protein 1	Nupr1	3.732131966
1457635_s_at	nuclear receptor subfamily 3, group C, member 1	Nr3c1	2
1459137_at	promyelocytic leukemia	Pml	2.462288827
1423986_a_at	shisa homolog 5 (Xenopus laevis)	Shisa5	2.29739671
1426538_a_at	transformation related protein 53	Trp53	2.828427125
1416926_at	transformation related protein 53 inducible nuclear protein1	Trp53inp1	3.249009585
1437277_x_at	transglutaminase 2, C polypeptide	Tgm2	8
1420499_at	GTP cyclohydrolase 1	Gch1	2.091825876
1419603_at	interferon activated gene 204///myeloid cell nuclear differentiation antigen	Ifi204	2.078920986
1450922_a_at	transforming growth factor, beta 2	Tgfb2	2.016764064
**Negative Regulation of Apoptosis**			
1418901_at	CCAAT/enhancer binding protein (C/EBP), beta	Cebpb	2.639015822
1425519_a_at	CD74 antigen	Cd74	3.249009585
1460251_at	Fas (TNF receptor superfamily member 6)	Fas	5.278031643
1420772_a_at	TSC22 domain family, member 3	Tsc22d3	3.482202253
1425927_a_at	activating transcription factor 5	Atf5	3.249009585
1451083_s_at	alanyl-tRNA synthetase	Aars	2
1449591_at	caspase 4, apoptosis-related cysteine peptidase	Casp4	9.18958684
1416693_at	forkhead box C2	Foxc2	2.143546925
1416983_s_at	forkhead box O1	Foxo1	2.143546925
1454958_at	glycogen synthase kinase 3 beta	Gsk3b	2.143546925
1438930_s_at	methyl CpG binding protein 2	Mecp2	2
1437122_at	B-cell leukemia/lymphoma 2	Bcl2	2.639015822
1422601_at	serine (or cysteine) peptidase inhibitor, clade B, member 9	Serpinb9	2.639015822
1426538_a_at	transformation related protein 53	Trp53	2.828427125

Results presented show genes with more than 2-fold increase in expression in 4T1 cells following contact co-culture with MSCs.

**Table 2:** Cluster of genes involved in the positive and negative regulation of proliferation.

ID	Gene Title			Gene Symbol	Fold Change
**Positive Regulation of Proliferation**					
1451021_a_at	Kruppel-like factor 5			Klf5	2.143546925
1418930_at	chemokine (C-X-C motif) ligand 10			Cxcl10	5.278031643
1426955_at	collagen, type XVIII, alpha 1			Col18a1	2.143546925
1416123_at	cyclin D2			Ccnd2	2.143546925
1422738_at	discoidin domain receptor family, member 2			Ddr2	2.828427125
1435888_at	epidermal growth factor receptor			Egfr	3.732131966
1448148_at	granulin			Grn	3.031433133
1423104_at	insulin receptor substrate 1			Irs1	2.143546925
1437303_at	interleukin 6 signal transducer			Il6st	2.29739671
1438930_s_at	methyl CpG binding protein 2			Mecp2	2
1419123_a_at	platelet-derived growth factor, C polypeptide			Pdgfc	4.59479342
1437122_at	B-cell leukemia/lymphoma 2			Bcl2	2.639015822
1454974_at	netrin 1			Ntn1	2.209770534
1437247_at	fos-like antigen 2			Fosl2	2.328358707
1450922_a_at	transforming growth factor, beta 2			Tgfb2	2.016764064
1417500_a_at	transglutaminase 2, C polypeptide			Tgm2	7.464263932
**Negative Regulation of Proliferation**					
1417394_at		Kruppel-like factor 4 (gut)		Klf4	3.031433133
1435479_at		bone morphogenetic protein 7		Bmp7	3.031433133
1440866_at		eukaryotic translation initiation factor 2-alpha kinase 2		Eif2ak2	4
1454693_at		histone deacetylase 4		Hdac4	2.828427125
1423754_at		interferon induced transmembrane protein 3		Ifitm3	2.639015822
1419665_a_at		nuclear protein 1		Nupr1	3.732131966
1437122_at		B-cell leukemia/lymphoma 2		Bcl2	2.639015822
1459137_at		promyelocytic leukemia		Pml	2.462288827
1417850_at		retinoblastoma 1		Rb1	2.143546925
1450165_at		schlafen 2		Slfn2	3.031433133
1430526_a_at		SWI/SNF related, matrix associated, actin dependent regulator ofchromatin, subfamily a, member 2		Smarca2	1.611863831
1416168_at		serine (or cysteine) proteinase inhibitor, clade F, member 1		Serpinf1	2.561671254
1426538_a_at		transformation related protein 53		Trp53	2.828427125
1450922_a_at			transforming growth factor, beta 2	Tgfb2	2.016764064

Results presented show genes with more than 2-fold increase in expression in 4T1 cells following contact co-culture with MSCs.

**Table 3:** Genes classified into oncogenes, tumor markers and tumor promoters.

ID	Gene Title	Gene Symbol		Fold Change
**Oncogene**				
1416268_at	E26 avian leukemia oncogene 2, 3′ domain		Ets2	2.462288827
1417558_at	Fyn proto-oncogene		Fyn	2.828427125
1423100_at	FBJ osteosarcoma oncogene		Fos	4.28709385
1426452_a_at	RAB30, member RAS oncogene family		Rab30	2.143546925
1450918_s_at	Rous sarcoma oncogene		Src	2
**Tumor Marker**				
1416612_at	cytochrome P450, family 1, subfamily b, polypeptide 1		Cyp1b1	2.462288827
1419330_a_at	glycoprotein A33 (transmembrane)		Gpa33	4
1419554_at	CD47 antigen (Rh-related antigen, integrin-associated signal transducer)		Cd47	2.143546925
1422567_at	family with sequence similarity 129, member A		Fam129a	6.964404506
1448380_at	lectin, galactoside-binding, soluble, 3 binding protein		Lgals3bp	3.249009585
1450264_a_at	choline kinase alpha		Chka	4
1451446_at	anthrax toxin receptor 1		Antxr1	4
1417185_at	lymphocyte antigen 6 complex, locus A		Ly6a	4.924577653
**Tumor Promoter**				
1418930_at	chemokine (C-X-C motif) ligand 10		Cxcl10	5.278031643
1419879_s_at	tripartite motif-containing 25		Trim25	2.143546925
1438629_x_at	granulin		Grn	2.29739671
1416693_at	forkhead box C2		Foxc2	2.143546925
1418910_at	bone morphogenetic protein 7		Bmp7	2.29739671
1423104_at	insulin receptor substrate 1		Irs1	2.143546925
1416016_at	transporter 1, ATP-binding cassette, sub-family B (MDR/TAP)		Tap1	6.964404506
1441026_at	poly (ADP-ribose) polymerase family, member 4		Parp4	3.249009585

Results presented show genes with more than 2-fold increase in expression in 4T1 cells following contact co-culture with MSCs.

## Discussion

MSCs within the tumor microenvironment exert multiple tumorigenic effects such as enhancement of tumor growth, metastasis, and angiogenesis [24]. In this study, we used murine MSCs and 4T1 mammary cancer cells to determine how MSCs affect tumor progression. MSCs enhanced 4T1 tumor growth and lung metastasis when co-injected into the mouse mammary fat pad (Fig. 1 and 3). MSCs significantly increased the tumorigenic potential of 4T1 cells *in* *vivo*. When co-injected with MSCs, only five or ten 4T1 cells could form orthotopic tumors.When co-cultured with MSCs, 4T1 cells had upregulated expression of several oncogenes and tumor promoting genes. We propose that MSCs are able to affect the cancer cells, thereby allowing them to become tumorigenic, possibly by modulating their gene expression. These results suggest that through modulation by MSCs, 4T1 cancer cells acquire higher self-renewal ability, which is an important characteristic of cancer stem cells. Other studies have shown that MSCs enhance the cancer stem cell population *in vitro* [25], [26]. These results indicate a high risk of breast cancer when performing cell therapy with MSCs.

Initiation is a crucial process in tumor formation. Subcutaneous tumors become palpable when the diameter reaches approximately 5 mm and at this time the cell number within the tumor could exceed 1×10^8^. Due to the limited resolution of clinical imaging, the early stage of tumorigenesis is difficult to detect. Fluorescence imaging using color-coded cells, together with an *in vivo* imaging system, is able to clearly visualize the morphological changes in cancer cells that occur during tumor progression, migration or interaction within the stroma at the single-cell level [27]. In addition to observation of the gross tumor, the imaging technology described in this report enables in vivo single-cell level visualization of response to cancer treatment.

In the present study, we co-injected either 5 or 10 RFP-4T1 cells and 10^5^ GFP-MSCs to monitor the process of 4T1 tumorigenesis *in vivo*. With longitudinal colored-coded fluorescence imaging, these few RFP-expressing 4T1 cells initially resided in the GFP-expressing MSC-mass and retained their viability, suggesting that MSCs create a microenvironment favoring cancer cell survival and eventual proliferation. The proliferation of RFP-expressing 4T1 cells increased over time and by day 15, the cancer cells had grown and were distributed within the MSC-mass.

The promoting effect of MSCs on tumor growth is related to increased tumor vessel formation [28]. Several factors secreted by MSCs are also known to influence angiogenesis, including FGF, MCP1, PDGF-α and VEGF [29]–[32]. Our results further provide the image-based evidence that MSCs create a vascularized microenvironment. Co-implantation of 10^5^ GFP-expressing MSCs in the mouse mammary fat pad produced a spherical mass of approximately 1 mm in diameter that was comprised mainly of MSCs after 6 days. Surrounding vessels increased and many of them were distributed within the MSCs mass. The vessels become thicker and increased in number over time (Fig. 6a, 6b and 6j). CD31, an endothelial marker, could also be detected to a greater extent in tumors derived from 4T1+MSCs than in tumors from 4T1 alone (Fig. 2e and 2f). These results demonstrated that MSCs increased tumor angiogenesis, suggesting that MSCs affect both cancer cells and the tumor microenvironment.

### Conclusions

Our results indicate that MSCs promote mammary tumorigenesis, tumor growth and metastasis, possibly by modifying cancer-related gene expression and generating a vascularized microenvironment. The above findings suggest that MSCs in the tumor microenvironment may be a potential target for developing strategies of cancer therapy in the future. Care must be taken when considering MSC therapy in patients with a high risk of breast cancer.
